# A Machine Learning Approach for Predicting Wage Workers’ Suicidal Ideation

**DOI:** 10.3390/jpm12060945

**Published:** 2022-06-09

**Authors:** Hwanjin Park, Kounseok Lee

**Affiliations:** 1 Department of Occupational & Environmental Medicine, Kangbuk Samsung Hospital, Sungkyunkwan University School of Medicine, Seoul 03181, Korea; ppakans@naver.com; 2 Department of Psychiatry, Hanyang University Medical Center, Seoul 04763, Korea

**Keywords:** worker, suicide, machine learning, prediction, depression, risk factors

## Abstract

(1) Background: Workers spend most of their days working. One’s working environment can be a risk factor for suicide. In this study, we examined whether suicidal ideation can be predicted using individual characteristics, emotional states, and working environments. (2) Methods: Nine years of data from the Korean National Health and Nutrition Survey were used. A total of 12,816 data points were analyzed, and 23 variables were selected. The random forest technique was used to predict suicidal thoughts. (3) Results: When suicidal ideation cases were predicted using all of the independent variables, 98.9% of cases were predicted, and 97.4% could be predicted using only work-related conditions. (4) Conclusions: It was confirmed that suicide risk could be predicted efficiently when machine learning techniques were applied using variables such as working environments.

## 1. Introduction

Adult workers spend most of their days working. Work is good for one’s mental health, but a poor work environment can cause physical and mental health problems [1]. When a worker has mental health problems, various difficulties such as low self-confidence, high tension, frequent mistakes, low energy, and conflicts between colleagues can occur, which can ultimately lead to a decrease in work performance. If mental health problems eventually lead to suicide, valuable personal and social assets will be lost not only to workers, but also to businesses. Therefore, management of the mental health of workers and suicide prevention should be carried out in the workplace.

The working environment refers to all factors that affect work activities, including various managerial factors, human relationships, and physical conditions related to job performance, as well as the physical environment in terms of job performance. In other words, the working environment can be divided into the physical environment, including the type of employment and type of business, the labor structure, which includes the number of employees, working hours, and shift work, and the socio-psychological environment, which is affected by the characteristics of the work being performed [2].

It was found that the longer the working hours, the higher was the risk of depression and suicidal ideation [3,4,5]. In contrast, working shorter than normal hours significantly reduced suicidal ideations, suggesting that priority working hours are a major risk factor [3]. The risk of absenteeism due to accidents and illness was three times higher in those who did not work more than 40 h a week [6]. Therefore, the working hours of workers can be an important factor in the working environment. Long working hours can pose a threat to the health and safety of workers, their families, businesses, and the public, even if they are voluntary choices made by workers. For this reason, the International Labor Organization suggests that the appropriate working hours are eight hours a day or 48 h a week [7].

In addition, irregular working environment factors, such as overtime and irregular working hours, can cause changes in an individual’s physiological rhythm and threaten their health [8]. In the case of nonregular workers, the rate of use of sick leave, which can be used in cases of illness, was significantly lower than that of regular workers [9]. Another study suggested that suicidal ideation was significantly higher among nonregular workers than among regular workers [10].

Job characteristics include job control and autonomy, and it is known that exposure to excessive noise, sunlight, and specific substances (e.g., pesticides) can affect the work environment [11]. It has also been shown that occupational skill level according to the type of occupation affects one’s suicide risk [12].

Although these characteristics can be used to evaluate the risk of suicide in a worker group, there have been no studies on the overall risk of several factors. Suicide does not occur due to a single cause; rather it occurs as a result of the complex contribution of several factors such as biological vulnerability, psychological state, and clinical characteristics [13].

Although the early identification and treatment of high-risk patients is a key suicide prevention strategy [14], high-risk patients are often not recognized by healthcare providers [15]. To overcome this issue, a suicide risk screening study using machine learning has been conducted [16,17,18]. Machine learning algorithms can change and improve as they are exposed to new data, and these detection patterns have many potential advantages over passively guided approaches to model specifications, particularly in terms of efficiency, complexity, and flexibility [19]. Machine learning techniques have the advantage of being able to investigate a wide range of complex associations between a large number of potential factors to generate algorithms that optimize predictions [20].

To date, no studies have applied machine learning techniques to predict suicide risk in workers. Therefore, in this study, the demographic characteristics, medical history, and personal lifestyle differences of a group with suicidal thoughts were first examined. Their emotional status, stress levels, subjective health status, and depression factors were then examined to determine whether there were differences. In addition, we aimed to determine how work characteristics such as occupational classification, working hours, and work type affect suicidal ideation. This study aimed to investigate whether the suicide risk of workers could be efficiently predicted using machine learning techniques based on working environments and emotional factors.

## 2. Materials and Methods

### 2.1. Study Population

This study used data from the Korean National Health and Nutrition Examination Survey (KHANES). Data from a total of 9 years (2007–2013, 2015, and 2017) were used [16]. In 2014, 2016, and 2018, suicidal ideation among adults was not investigated, and part-time work (full-time or part-time) was not investigated in 2019; therefore, these types of data were excluded from the study. The KHANES is a national survey of the health and nutritional status of noninstitutionalized civilians in Korea [21]. This study targeted 12,865 wage earners aged 19 years or older from nine years of data. The subjects were administered a questionnaire that included the question: “During the past year, have you ever felt that you were willing to die?” Responses were acquired from 12,816 people, excluding 49 who did not respond. This study was approved by the Institutional Review Board of the Korea Disease Control and Prevention Agency (IRB No 2018-01-03-P-A).

### 2.2. Work-Related Conditions

Occupations were classified according to the major classification codes of Korea’s standard occupational classification system. Among the KHANES data, the work-related variables commonly investigated in the 9 years of data were as follows:

- Occupation type: (1) managers, experts, and related workers; (2) office workers; (3) service and sales workers; (4) skilled workers in agriculture, forestry, and fisheries; (5) technicians, equipment operators, machine assembly and operation workers; (6) simple labor workers.
- Occupational status: (1) full-time; (2) temporary; (3) day worker.
- Working hour system: (1) full-time worker; (2) part-time worker.
- Type of shiftwork: (1) day work; (2) evening shift (14:00~24:00); (3) night shift (21:00–08:00); (4) regular day and night shift work; (5) 24 h shift work, split work (more than two working hours per day), irregular shift work, and other forms of shift work.
- Working hours: average working hours per week.
- Classification of regular and nonregular workers, which are thought to have an impact on suicidal ideation, were excluded as the survey was conducted only at 13, 15, and 17 years.

### 2.3. Data Processing and Machine Learning

This study evaluated the suitability of the suicidal ideation prediction model for wage workers by selecting 23 analyzable variables, presented in Table 1, that are thought to be related to suicidal ideation. For the second model, we conducted an analysis that included only work-related variables, including occupations, occupational status, working hour system, type of shift work, and weekly working hours. We analyzed whether suicidal ideation could be predicted using only the work-related conditions of wage workers.

Among machine learning algorithms, random forest (RF) is a method for finding optimal results through randomly combining results and constructing several decision trees. Random forest is widely used due to its good predictive performance, especially in mental health-related studies [16,22]. Therefore, in this study, a RF was constructed to evaluate various variables, work-related variables, and the suitability of the suicidal ideation prediction model for wage workers.

The subjects were analyzed by dividing the training and test data by 70% and 30%, respectively. Repeated 10-fold cross-validation was used to develop the analysis model. In the 10-fold cross validation, the data is divided into 10 folds of the same size, and the remaining 9 folds are used for training while each fold is used as a validation set. This is repeated five times to avoid overfitting and to generalize the prediction model. To find the optimal parameter of the RF, the analysis was conducted with the grid search method and the optimized hyperparameter of the RF was applied to the final model. The fitted model was used to predict the suicidal ideation of wage workers and it was compared with actual suicidal ideation using a confusion matrix with the training data. The performance of the predictive model was evaluated by calculating the accuracy, sensitivity, specificity, positive predictive value, negative predictive value, and area under the curve. Area under the curve (AUC) means the area under the roc curve drawn with a false positive rate as the x-axis and true positive rate as the y-axis. An AUC value of 0.5 indicated no discriminative value. An AUC value ≥0.75 is considered clinically useful [23]. R version 4.1.2 was used for all analyses with the “mice” package in R for processing missing data [24], the “caret” package for cross validation, and the “ROCR” package for the ROC curve.

## 3. Results

### 3.1. Differences in General Characteristics of Workers according to Suicidal Ideation and Work-Related Conditions

A suicide-related questionnaire was administered to 12,816 subjects, and it included the question: “During the past year, have you ever felt that you were willing to die?” A total of 915 people answered yes (7.14% of the subjects). The proportion of women who had suicidal thoughts in the past year was 66.7%, which was higher than that of women who had not (47.3%) (*p* < 0.001). The lower the household income (*p* < 0.001) and the lower the education level, the higher the rate of suicidal ideation (*p* < 0.001). Subjects in the suicidal ideation group were divorced (7.3%), separated, or widowed (9.1%), and the ratios of these factors were higher than those in the nonsuicidal group. Marital status was also significantly related to suicidal ideation (*p* < 0.001). Hypertension (*p* = 0.165), stroke (*p* = 0.469), cancer (*p* = 0.626), and diabetes (*p* = 0.994) were not associated with suicidal ideation, but hyperlipidemia (11.3%, *p* = 0.038), myocardial infarction, and angina pectoris (2.2%, *p* = 0.023) were, and the rates of these in the suicidal ideation group was significantly higher than that in the group without suicidal ideation. In the suicidal ideation group, the proportion (8.4%) of the group who drank more than four times a week was higher than that of the group without suicidal thoughts (5.8%, *p* < 0.001), and smoking (*p* = 0.049) was significantly associated with suicidal ideation. In the suicidal ideation group, the suicidal ideation rate was significantly higher in the group who felt extreme stress in their daily life (16.8%, *p* < 0.001). Bad (26.5%) or very bad (5.1%) subjective health status reports were also significantly higher in the suicidal group (*p* < 0.001). The rate of experiencing depressive symptoms lasting more than two weeks was overwhelmingly higher in the suicidal group (51.3%) compared to in the nonsuicidal group (7.1%) (*p* < 0.001), and the lower the EQ-5D score, the higher the rate of suicidal thoughts. This ratio was also significantly high (*p* < 0.001). BMI was not significantly associated with suicidal ideation (*p* = 0.395).

Regarding work-related conditions, in the suicidal ideation group, simple labor workers had the highest rate of suicidal thoughts (30.8%). In the nonsuicidal group, the proportion of officials, experts, and related workers was highest at 25.7% (*p* < 0.001). In the group with suicidal thoughts, the ratio of regular workers was lower and the ratio of temporary and daily workers was higher than in the group without suicidal thoughts (*p* < 0.001). The proportion of part-time workers was significantly higher in the suicidal ideation group (26.0%) than in the nonsuicidal group (19.1%, *p* < 0.001). In the suicidal ideation group, the proportion of shift workers was higher, as was the ratio of evening shift workers (9.8%) to night shift workers (3.1%; *p* < 0.001). Working hours per week were shorter in the group with suicidal thoughts (38.97 ± 17.65) than in the group without suicidal ideations (41.25 ± 15.74, *p* < 0.001, Table 1).

### 3.2. Prediction Model—Random Forest

In this study, a machine learning technique was used to build a model to predict suicidal ideation among adult wage workers. After analyzing the predictive model for suicidal ideations related to all variables, including depressive symptoms, the predictive model for suicidal ideations related to working conditions, including only the variables related to working conditions, was analyzed. Random forest analysis was performed on both the training data and test data, and the results for the training data are presented. The AUC of the random forest model, which included all variables, including depressive symptoms, was 0.922, and when only work-related conditions were analyzed, the AUC was 0.818, showing good performance. When looking at the confusion matrices, when all variables were included, 100 people out of 641 people who actually had suicidal thoughts were predicted, and 8331 people who did not have suicidal thoughts were accurately predicted. Thus, the accuracy of the model was 0.989, the sensitivity was 0.844, the specificity was 1.000, the positive predictive value was 1.000, and the negative predictive value was 0.988 (Table 2).

When analyzing only work-related conditions, 408 out of 641 people who actually had suicidal thoughts were predicted, and all 8331 people who did not have suicidal thoughts were predicted. Thus, the accuracy of the model was 0.974, the sensitivity was 0.637, the specificity was 1.000, the positive predictive value was 1.000, and the negative predictive value was 0.973 (Table 3).

## 4. Discussion

This study predicts suicidal ideation in adult wage workers using work-related indicators, working environment factors, emotional state information, and so on. Compared with the group without suicidal thoughts, the participants with suicidal thoughts had higher household incomes, stress levels, and symptoms of depression. They were also more likely to smoke and have chronic diseases. In terms of working environment, it was found that simple, part-time, and shift workers had higher suicidal thoughts. When suicidal ideation was predicted using all these independent variables, 98.9% of subjects with suicidal thoughts could be predicted, and 97.4% could be predicted using only work-related conditions. Depression was the biggest contributing factor to suicidal ideations, and it is known that the global labor loss due to emotional problems is $2.5 trillion, aggravating the economic loss of companies [25].

The work environment acts as a stress factor during work and can lead to depression and suicide; therefore, management of job stress is important to lower job-related suicide [26]. Working environment variables, such as long hours and shift work, can cause major job stress. Such job stress increases corticosteroids in the body, and the continuous increase in corticosteroids directly affects the brain and inhibits the regulation of emotions by downregulating glucocorticoid receptors, making workers vulnerable to depression. This may lead to suicide through direct or indirect complex actions involving other factors [27]. This study confirmed that occupational status, working hours, and working type were all affected. Although unavoidable circumstances are inevitable, in the case of changeable environmental factors, the emotional health of workers can be improved by establishing legal standards.

Although it has been reported that depression and suicide risk increase when working hours are high [3,4], in this study, when compared to the average working hours, the working hours of the group at risk of suicide were relatively short. First, suicidal ideation appears to be related to the significantly lower job stability in the group with suicidal thoughts [28]; the proportion of temporary or daily workers with low job security was 43% (28% in the nonsuicidal group). Second, a previous study showed a significant increase in depression when working hours were extremely long, and suicidal ideation was also found to increase when working more than 69 h [4]. These points are different from the results of this study.

Quality of life in terms of personal health is an indicator of one’s health. It is related to an individual’s health in terms of physical, emotional, and social aspects. Notably, quality of life was not good in the group with suicidal thoughts [29]. Although subjective, decreased overall life satisfaction may be a risk factor for suicidal ideations. In addition, in the group with suicidal thoughts, the perception of stress was relatively high: they felt more depressed for more than 2 weeks, and they felt that their subjective health was not good. Mental health problems such as worker depression can lead to problems such as absenteeism, decreased and exhausted work motivation, tension and relationship conflicts among colleagues, increased accidents at work, and decreased productivity in companies [30]. For workers, depression eventually contributes to long-term absenteeism and early retirement [31,32]. It was also found that workers with major depressive disorder lost 33.4% of their annual salary as a result of decreased productivity due to absenteeism and presenteeism, whereas the control group lost 2.5% of their annual salary [33]. As such, workers’ depression can affect not only their personal health but also lower corporate productivity and, ultimately, lower national competitiveness.

When analyzing by occupation, it was confirmed that the simple labor workers had a relatively high suicidal ideation rate. In a previous study, suicidal ideation rates according to the job status of wage workers were 2.0%, 5.0%, and 7.0% for regular, temporary, and daily workers, respectively. For men, the rate of suicidal ideation was the highest among daily workers (9.6%), and for women, it was the highest among temporary workers (5.6%) [34].

Work-related difficulties, such as excessive job demands, relationship conflicts, and emotional labor, can increase job stress, job satisfaction, emotional exhaustion, and negative health symptoms, such as depression [35,36]. However, factors influencing suicide among workers may include personal life stress and individual sensitivity, in addition to the working environment and job stress.

Suicide prevention programs in the workplace should be structured to prevent each of these stressors and enable the early detection and treatment of suicidal ideations [37]. This study confirmed the possibility of predicting suicidal ideation using common variables and work environments. Among other things, it is necessary to understand the mental health of workers with depression or suicidal thoughts. Once issues have been identified, it can be helpful to take measures that are suitable for each worker or to improve workplace environmental factors.

The limitations of this study are as follows: First, only cross-sectional factors were used, and an overall evaluation of work environments was not performed. In situations where it is impossible to evaluate various working environments with a single evaluation index, it will be helpful to index the work environment by evaluating these environmental aspects through indicators that can be evaluated in the future. Second, only the cross-sectional area evaluation results were used. In the future, it will be necessary to confirm these findings via a cohort study of workers. Third, only suicidal ideation was evaluated. Suicidal behavior can be divided into suicidal thoughts, plans, and attempts. In the end, suicidal thoughts can lead to suicide plans and even suicide attempts, but it should be taken into account that not all suicidal thoughts lead to suicide attempts. Meanwhile, studies using machine learning or artificial intelligence to predict suicide have been conducted. However, these methods may be limited by the quality of the input data, and their clinical utility has not yet been established [18].

The prediction of suicide using machine learning is mainly based on the results of suicide-related questionnaires or emotional evaluations [16,38]. However, the acquisition of such evaluation data may be difficult in practice, as disadvantages in work may be expected if it is targeted to actual workers. The strength of this study is that it utilized typical variables and large-scale data obtained through conventional investigations. This is a national project, and a similar type of investigation will be conducted in the future, making it possible to repeat and improve learning through open data. As for the variables used in the study, evaluation results from environmental and simple emotional aspects were used as opposed to special measurements or evaluations. Above all, this study has great significance in that it predicts the risk of suicide by synthesizing factors such as work-related environmental variables and the emotional aspects of workers. In the future, it will be possible to select risk factors through large-scale learning using various risk factors and create algorithms that utilize factors that characterize the individual status of workers, such as their language, facial expressions, or social media data.

## 5. Conclusions

This study attempted to predict suicide accidents among wage workers using variables from the National Health and Nutrition Examination Survey. When suicidal ideation cases were predicted using 23 work-, life-, and emotion-related variables with the random forest method, 98.9% were predicted more accurately (F1 score was 0.915), and 97.4% could be predicted using only work-related variables. It was confirmed that when the machine learning technique was applied to workers using working environment factors, suicide could be efficiently predicted. In the future, it will be necessary to identify risk factors using various variables and to make efforts to protect the emotional health of workers.

## Figures and Tables

**Table 1:** General characteristics of subjects according to suicidal ideation and work type.

		With Suicidal Ideation (*n* = 915)	Without Suicidal Ideation (*n* = 11,901)	*p* Value
Sex	Male	305 (33.3%)	6276 (52.7%)	<0.001
	Female	610 (66.7%)	5625 (47.3%)	
Age		45.12 ± 15.34	43.96 ± 13.79	0.014
Family income	Lower	192 (21.0%)	1169 (9.8%)	<0.001
	Low-intermediate	209 (22.8%)	2159 (18.1%)	
	Middle	212 (23.2%)	2736 (23.0%)	
	Upper-intermediate	158 (17.3%)	2870 (24.1%)	
	Upper	144 (15.7%)	2967 (24.9%)	
Education	Lower than elementary school	200 (21.9%)	1348 (11.3%)	<0.001
	Middle school	113 (12.3%)	979 (8.2%)	
	High school	324 (35.4%)	4150 (34.9%)	
	Higher than university	278 (30.4%)	5424 (45.6%)	
Marriage status	Unmarried	232(25.4%)	2566 (21.6%)	<0.001
	Married	533 (58.3%)	8384 (70.4%)	
	Divorced	67 (7.3%)	426 (3.6%)	
	Other	83 (9.1%)	525 (4.4%)	
Hypertension history	No	771 (84.3%)	10,226 (85.9%)	0.165
	Yes	144 (15.7%)	1675 (14.1%)	
Dyslipidemia history	No	812 (88.7%)	10,808 (90.8%)	0.038
	Yes	103 (11.3%)	1093 (9.2%)	
Stroke history	No	904 (98.8%)	11,787 (99.0%)	0.469
	Yes	11 (1.2%)	114 (1.0%)	
Myocardial infarction or angina history	No	895 (97.8%)	11,748 (98.7%)	0.023
	Yes	20 (2.2%)	153 (1.3%)	
Diabetes mellitus history	No	870 (95.1%)	11,315 (95.1%)	0.994
	Yes	45 (4.9%)	586 (4.9%)	
Cancer history	No	896 (97.9%)	11,624 (97.7%)	0.626
	Yes	19 (2.1%)	277 (2.3%)	
Alcohol consumption	Not a drinker	182 (19.9%)	2110 (17.7%)	<0.001
	One or less/month	183 (20.0%)	2187 (18.4%)	
	One time/month	112 (12.2%)	1360 (11.4%)	
	2~4 times/month	210 (23.0%)	3377 (28.4%)	
	2~3 times/week	151 (16.5%)	2181 (18.3%)	
	4 or more/week	77 (8.4%)	686 (5.8%)	
Smoking	Daily	228 (24.9%)	2560 (21.5%)	0.049
	Occasional	24 (2.6%)	361 (3.0%)	
	Not a smoker	663 (72.5%)	8980 (75.5%)	
Subjective stress	Very much	154 (16.8%)	375 (3.2%)	
	Many	426 (46.6%)	2681 (22.5%)	
	Little	310 (33.9%)	7413 (62.3%)	
	Rarely	25 (2.7%)	1432 (12.0%)	
Subjective health status	Very good	20 (2.2%)	572(4.8%)	<0.001
	Good	171 (18.7%)	3709 (31.2%)	
	Moderate	434 (47.4%)	6197 (52.1%)	
	Bad	243 (26.5%)	1306 (11.0%)	
	Very bad	47 (5.1%)	117 (1.0%)	
Depressed mood over 2 weeks	No	446 (48.7%)	11,059 (92.9%)	<0.001
	Yes	469 (51.3%)	842 (7.1%)	
EQ5D score		0.91 ± 0.12	0.97 ± 0.07	<0.001
Body mass index		23.64 ± 3.75	23.67 ± 3.42	0.395
Occupation	Managers, experts, and related workers	184 (20.1%)	3061 (25.7%)	<0.001
	Office worker	153 (16.7%)	2586 (21.7%)	
	Service and salespersonnel	183 (20.0%)	1952 (16.4%)	
	Skilled workers inagriculture, forestry, and fishing	2 (0.2%)	62 (0.5%)	
	Technician, device/machine operator, and assembly worker	111 (12.1%)	1995 (16.8%)	
	Simple labor worker	282 (30.8%)	2245 (18.9%)	
Occupational status	Full-time employee	519 (56.7%)	8679 (72.9%)	<0.001
	Temporary	227 (24.8%)	2149 (19.1%)	
	Day job	169 (18.5%)	1073 (9.0%)	
Working hour system	Full-time	677 (74.0%)	9629 (80.9%)	<0.001
	Part-time	238 (26.0%)	2272 (19.1%)	
Shiftwork type	Day shift	725 (79.2%)	9806 (82.4%)	<0.001
	Evening shift (14:00~24:00)	90 (9.8%)	810 (6.8%)	
	Night shift (21:00~8:00)	28 (3.1%)	228 (1.9%)	
	Regular day and nightshift work	36 (3.9%)	550 (4.6%)	
	Etc.	36 (3.9%)	507 (4.3%)	
Weekly working hours		38.97 ± 17.65	41.25 ± 15.74	<0.001

Values are presented as *n* (%) or mean ± SD. EQ-5D: EuroQoL-5D.

**Table 2:** Confusion matrix and prediction scores of random forest model with all variables.

Observation	Prediction (*n*)		Sensitivity	Specificity	Accuracy	Positive Predictive Value	Negative Predictive Value	F1 Score	AUC
	Yes	No	(%)	(%)	(%)	(%)	(%)		
Yes	541	100	84.4	100	98.9	100	98.8	0.915	0.922
No	0	8331							

**Table 3:** Confusion matrix and prediction scores of random forest model with work-related variables.

Observation	Prediction (*n*)		Sensitivity	Specificity	Accuracy	Positive Predictive Value	Negative Predictive Value	F1 Score	AUC
	Yes	No	(%)	(%)	(%)	(%)	(%)		
Yes	408	233	63.65	100	97.4	100	97.3	0.778	0.818
No	0	8331							

## Data Availability

Not applicable.
